# The Positive and Negative Immunoregulatory Role of B7 Family: Promising Novel Targets in Gastric Cancer Treatment

**DOI:** 10.3390/ijms221910719

**Published:** 2021-10-03

**Authors:** Nadia Bolandi, Afshin Derakhshani, Nima Hemmat, Amir Baghbanzadeh, Zahra Asadzadeh, Mina Afrashteh Nour, Oronzo Brunetti, Renato Bernardini, Nicola Silvestris, Behzad Baradaran

**Affiliations:** 1 Immunology Research Center, Tabriz University of Medical Sciences, Tabriz 516615731, Iran; nbolandi@yahoo.com (N.B.); afshin.derakhshani94@gmail.com (A.D.); nima.hemmat1995@gmail.com (N.H.); amirbaghbanzadeh@gmail.com (A.B.); Zahraasadzadeh2834@gmail.com (Z.A.); mina_afrashteh@yahoo.com (M.A.N.); 2 Department of Biochemistry, Faculty of Medicine, Urmia University of Medical Sciences, Urmia 571478334, Iran; 3 Laboratory of Experimental Pharmacology, IRCCS Istituto Tumori Giovanni Paolo II, 70124 Bari, Italy; 4 Medical Oncology Unit—IRCCS Istituto Tumori “Giovanni Paolo II” of Bari, 70124 Bari, Italy; dr.oronzo.brunetti1983@gmail.com; 5 Department of Biomedical and Biotechnological Sciences, University of Catania, Via S. Sofia 97, 95100 Catania, Italy; bernardi@unict.it; 6 Department of Biomedical Sciences and Human Oncology (DIMO), University of Bari, 70124 Bari, Italy; 7 Department of Immunology, Faculty of Medicine, Tabriz University of Medical Sciences, Tabriz 516615731, Iran; 8 Pharmaceutical Analysis Research Center, Tabriz University of Medical Sciences, Tabriz 516615731, Iran

**Keywords:** B7 family, immune checkpoints, immunotherapy, gastric cancer

## Abstract

Gastric cancer (GC), with a heterogeneous nature, is the third leading cause of death worldwide. Over the past few decades, stable reductions in the incidence of GC have been observed. However, due to the poor response to common treatments and late diagnosis, this cancer is still considered one of the lethal cancers. Emerging methods such as immunotherapy with immune checkpoint inhibitors (ICIs) have transformed the landscape of treatment for GC patients. There are presently eleven known members of the B7 family as immune checkpoint molecules: B7-1 (CD80), B7-2 (CD86), B7-H1 (PD-L1, CD274), B7-DC (PDCD1LG2, PD-L2, CD273), B7-H2 (B7RP1, ICOS-L, CD275), B7-H3 (CD276), B7-H4 (B7x, B7S1, Vtcn1), B7-H5 (VISTA, Gi24, DD1α, Dies1 SISP1), B7-H6 (NCR3LG1), B7-H7 (HHLA2), and Ig-like domain-containing receptor 2 (ILDR2). Interaction of the B7 family of immune-regulatory ligands with the corresponding receptors resulted in the induction and inhibition of T cell responses by sending co-stimulatory and co-inhibitory signals, respectively. Manipulation of the signals provided by the B7 family has significant potential in the management of GC.

## 1. Introduction

Gastric cancer (GC) is classified as the third leading cancer in cancer-related mortality and the fifth most widespread malignancy worldwide. Based on GLOBOCAN 2018 data, the GC prevalence rate tends to be higher in East Asia compared to Western countries [1]. GC patients do not show any particular symptoms in the early stages of cancer, and diagnosis of GC is delayed. Therefore, although the mortality rate of GC has been declining in recent years, it is still considered life-threatening cancer [2]. The combination of some chemotherapy drugs, such as fluoropyrimidine with platinum base and irinotecan with taxane and surgical treatment, can be used as a standard option to treat patients with GC. However, the average survival rate of these patients is less than 10 months [3]. Immunotherapy is well studied as an efficient strategy for cancer therapy due to the induction of specific and long-lasting anti-cancer effects. Immunotherapy via ICIs is considered as a novel approach for cancer treatment and can prevent recurrence of tumors [4]. The immune system is frequently suppressed as tumors progress, interfering with a successful anti-tumor response. On the other hand, in autoimmune diseases, the immune system actively attacks and destroys the self-tissues. In order to elicit protective immunity against cancer and infection and inhibit the overactivity of the immune system, immune responses need to be strictly controlled by the B7 family members, which contain co-stimulatory and co-inhibitory molecules [5,6]. T cells are introduced as central elements of adaptive immunity, and their activation depends on two signals: signal 1, or antigen recognition, where peptides presented by the main histocompatibility complex (MHC) are identified by T cell receptors (TCRs), and signal 2, or co-stimulation, involving the combination of co-regulators such as B7 proteins, consisting of co-stimulatory and co-inhibitory molecules expressed on antigen-presenting cells (APC). Co-stimulation is balanced by co-inhibitory signals, which finally describe whether the T cell response is activating or inhibitory [7]. The B7 family is one of the most significant secondary signaling mechanisms and is vital in preserving the balance between immune potency and the suppression of autoimmunity [8]. At present, the growing B7 family includes 11 members, which are B7-1 (CD80), B7-2 (CD86), B7-H1 (PD-L1, CD274), B7-DC (PDCD1LG2, PD-L2, CD273), B7-H2 (B7RP1, ICOS-L, CD275), B7-H3 (CD276), B7-H4 (B7x, B7S1, Vtcn1), B7-H5 (VISTA, Gi24, DD1α, Dies1 SISP1), B7-H6 (NCR3LG1), B7-H7 (HHLA2), and Ig-like domain-containing receptor 2 (ILDR2) [7,9,10]. It is reported that B7 molecules produce essential positive signals to start and support T-cell activity; moreover, they provide negative signals to regulate and stop T-cell reactions [11]. Additionally, the members of the B7 family appear as substantial elements in the regulation of tumor development, invasion, metastasis, and drug sensitivity, independent of the immune system [12]. Due to the operative function of ICIs in cancer treatment, the B7 family of molecules has extended remarkable consideration in recent years. In this review article, we describe the structure, expression, and function of the B7 family members. In addition, we focus on the role of the B7 family in humans’ GC and the potential targeting of B7 family members with monoclonal antibodies, which is a promising strategy for GC treatment.

## 2. B7-1 (CD80) and B7-2 (CD86)

**Figure 1:**
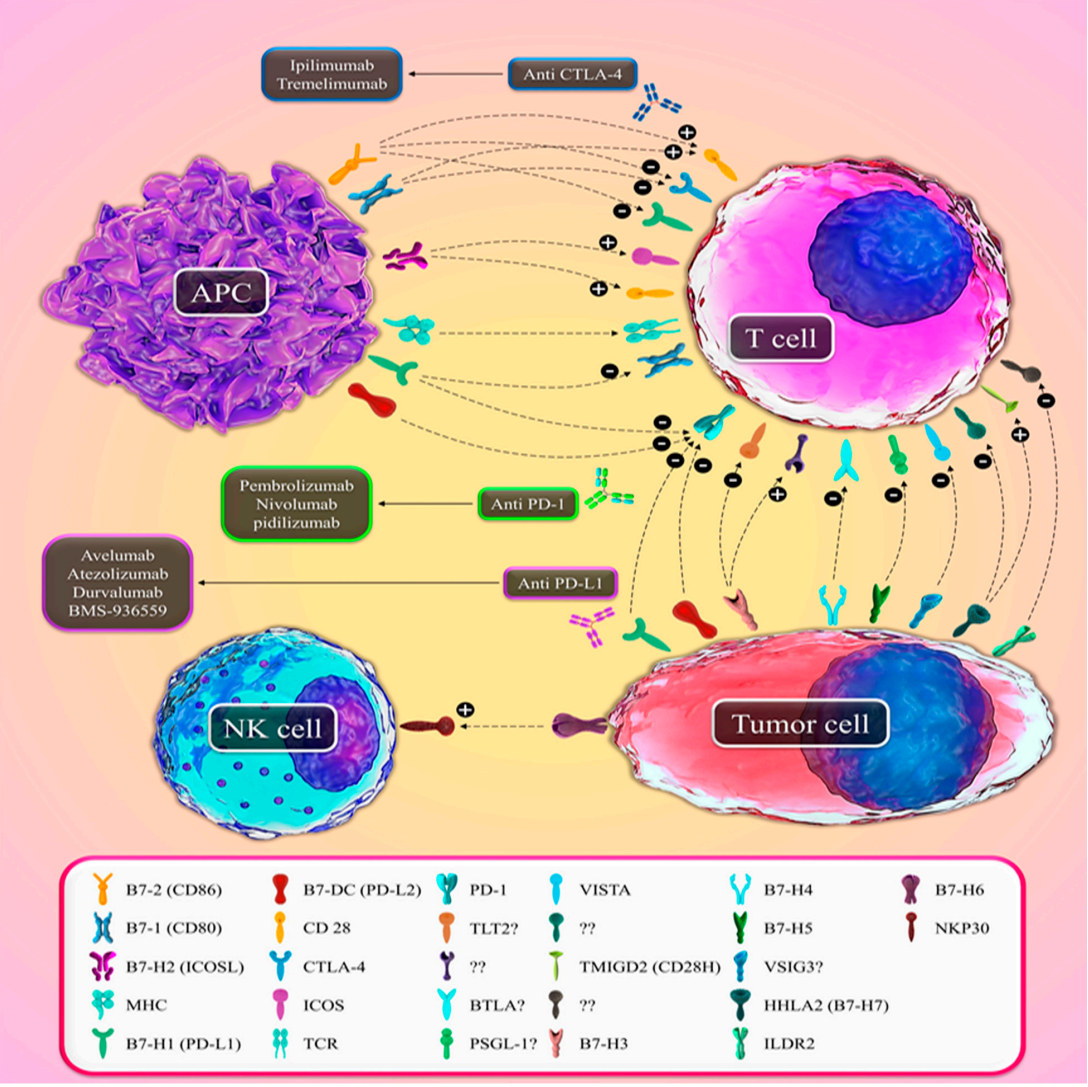
A suggested model for the functions of B7 family ligands with their cognate receptors. The interaction of the B7-1 and B7-2 with CD28, ICOSL with ICOS, and HHLA2 with TMIGD2 resulted in sending co-stimulatory signals. The interaction of NKP30 expressed in NK cells with B7-H6 expressed in tumor cells resulted in the activation of T cells or NK cells. The interaction of B7-1 and B7-2 with CTLA-4, PD-L1, and PD-L2 with PD-1, and PD-L1 with B7-1, provide co-inhibitory signals to stop T-cell reactions. The corresponding receptors that are generally accepted for B7-H3, B7-H4, B7-H5, and ILDR2 have not been reported.

Two identified members of the B7 family proteins, B7-1 (CD80) and B7-2 (CD86), could bind to CD28 and Cytotoxic T-Lymphocyte Antigen 4 (CTLA-4) and act as their ligands (Figure 1, Table 1). B7-1/2 ligands with similar structures and approximately 25 percentage homologies are APC-related (type I) surface proteins [13]. Both B7-1 and B7-2 consist of one Ig variable-like (IgV) domain and one Ig constant-like (IgC) domain. Despite their similar structure, B7-2 exists in the monomeric form on the surface of cells disparate from B7-1 [14]. B7-1 and B7-2 are expressed in dendritic cells, B/T cells, and APCs, and it is reported that the expression of B7-2 is up-regulated by APCs in stimulatory conditions [15,16]. B7-1 and B7-2 are different in the promotion and initiation of the T-cell responses. In this regard, the T-cell responses could be initiated and promoted by the expression of B7-2 and B7-1, correspondingly [16,17]. CTLA-4, the first-studied co-inhibitory molecule of the B7 family, was introduced as an initial receptor of the immune checkpoints [18]. CTLA-4 also defined CD152 as a homolog of CD28 (about 20 percent), sharing the identity of 30% and 27% of the level of amino acid in humans and murine, respectively. The structure of CTLA-4 includes domains and a leader peptide. These domains are the cytoplasmic tail, extracellular V domain, and ligand-binding domain, which have different numbers of amino acids [19]. CTLA-4/CD28 receptors are located on chromosome 1 at band C of mice, while they are located on chromosome 2 at bands q33-q34 of humans [20,21]. APCs, activated effector T-cells (Teffs), and regulatory T cells (Treg cells) express CTLA-4. Likewise, CTLA-4 is expressed on normal or neoplastic cells [22]. CD28 expression in humans occurs on 50% and 80% of CD8+ and CD4+ T-cells (individually). Additionally, it is proved that CD28 expression takes place on various cells containing plasma cells, bone marrow stromal cells, and neutrophils [23]. The binding of CTLA-4/CD28 to CD80 or CD86 ligands occurs at the immunological synapse between antigen-presenting cells (APCs) and T cells [24]. According to studies, CD28 strongly binds to B7-2 while CTLA-4 has a higher binding affinity to both B7-1 and B7-2 [25,26]. The interaction of B7-1 and B7-2 ligands with CD28 delivers stimulation signals to immune response and induces survival of T lymphocytes. The interaction of CTLA-4 with these ligands delivers inhibitory signals for activation of T-lymphocytes. It also results in decreased production of cytokines and immune responses against malignant tumors [27,28]. The inhibitory function of CTLA-4 is suppressed using humanized monoclonal antibodies such as ipilimumab and tremelimumab. These anti-CTLA-4 antibodies have been accepted to manage cancers such as GC, non-small cell lung cancer (NSCLC), and melanoma [29,30]. It is demonstrated that transfection of B7-1 as a co-stimulatory factor induces an immunogenicity mechanism to suppress lymph node metastasis in GC. Moreover, it has been proven that the expression of B7-1 is decreased in GC and thus, increased expression of B7-1 on gastric GC cells decreases tumor growth. Therefore, B7-1, due to its anti-tumor functions, can be utilized in immunotherapy of GC [31,32]. Following previous findings, the prognostic role of B7-1 is detected in GC patients. It is indicated that B7-1 is expressed in low levels in the tissue of GC in comparison with normal gastric tissue. Moreover, it is reported that poor disease-free survival (DFS) and overall survival (OS) and immune evasion in GC result from the downregulation of B7-1. Thus, B7-1 might be considered an efficient target for GC therapy [33]. Interestingly, in contrast to the above results, Yang and colleagues reported that the protein levels of B7-1, B7-2, CTLA-4, and CD28 are broadly expressed in GC tissues compared to normal tissues adjacent to the tumors. They revealed that B7-1, B7-2, CTLA-4, and CD28 expression could induce tumor angiogenesis in GC by activating VEGF-A expression. Consequently, for assessing the biological behaviors, CD28, CTLA-4, B7-1, B7-2, and VEGF-A expression levels might be beneficial in GC [34].

## 3. B7-H2 (ICOSL)

The other co-stimulatory ligand that binds to the CD28 family is B7-H2, also known as ICOSL and B7h. ICOSL as B7 homolog binds not only to the Inducible Co-Stimulator (ICOS) but also to the CD28 in humans (Figure 1, Table 1) [35,36]. ICOSL consists of an IgV–IgC extracellular domain. The encoding gene of ICOSL is located on the 21st and 10th chromosomes in humans and mice, respectively. The encoding gene of ICOS with five exons is situated on chromosome 2q33–34 [37]. The engagement of CD28 and TCR resulted in ICOS induction, and positive co-stimulatory signals were delivered [38]. Even though the ICOS expression is boosted on activated T-lymphocytes, it is diminished by unstimulated naïve T-cells. Additionally, ICOSL is chiefly expressed by various cells of APCs such as endothelial cells, macrophages, non-hematopoietic cells, B-cells, monocytes, and dendritic cells, but is not expressed via innate lymphoid cells or T lymphocytes [39]. Moreover, ICOSL is expressed at low levels in some types of solid tumors such as glioma, colorectal cancer, and GC [40]. The expression of this ligand on endothelial cells enhances both the activation of CD8+ memory T-cell and co-stimulation of CD8+ T-cell [41,42]. ICOS and CD28 receptors have different roles in contrast to CTLA-4. These members of the B7 family, via competitive anti- and pro-inflammatory effects, regulate the immune system’s response [43]. As a consequence of ICOSL and its relevant receptor interaction, the Bcl6 transcription factor is induced to form follicular helper T cells [44,45]. Activating T-cells requires the activation of phosphatidylinositol-3-kinase (PI3K), which correlates with signal transduction pathways and induces the Th2 pathway [46]. Additionally, depending on the cell type, these interactions have an increasing effect in the production of several T cell cytokines and an inhibitory effect on apoptosis by increasing the activity and function of intracellular molecules [47,48]. One of the essential mechanisms related to the ICOS/ICOSL pathway is the induction of anti-tumor response mechanisms such as stimulation of CD8+ T cell response [49]. In contradiction to this role, ICOS decreases anti-tumor immunity because of the increased function of Treg cells, mainly through the secretion of IL10 and TGF-β [50]. Preliminary results from human trials of INDUCE-1 (NCT02723955), as an ICOS agonist antibody, showed that INDUCE-1 individually or in combination with PD-1 blocking antibodies such as pembrolizumab plays an essential role in cancer patients [51]. Nagase et al. demonstrated that ICOS+, Foxp3, and CD4+ T cells were plentifully found in tumor-infiltrating lymphocytes (TILs) in the end-stage of GC. They proved that ICOS expression in Foxp3 cells could be related to ICOSL, *Helicobacter pylori* infection, and TLR9 in plasmacytoid DCs (pDCs). The existence of the *Helicobacter pylori* antibody is a reason for considerable numbers of ICOS+ Foxp3+ cells in GC tissues. The results of their study demonstrated that immunotherapy agents, which target the ICOS checkpoint pathway and the eradication of *Helicobacter pylori*, are used as immunotherapy for GC [52]. Huang X-M et al. indicated that the number of ICOS+ Tregs is enhanced in peripheral blood (PB) of GC patients. There is a direct relation between ICOS+ Tregs number and illness severity. ICOS+ Tregs are generated from CD4+naïve T cells due to the increasing of pDCs number in GC. Therefore, pDCs and ICOS+ Tregs are involved in the suppression of immune response in GC [53]. MiR-24 has a leading role not only in regulating genes involved in various cancers but also in the oncogenesis of colorectal cancer, GC, etc. [54,55]. In this regard, Yang et al. demonstrated that miR-24 could regulate ICOSL expression. miR-24 also has an inhibitory effect on the expression of ICOSL by binding to the 3′-untranslated region (3′-UTR) of ICOSL. They found that single nucleotide polymorphisms (SNP) rs4819388, situated in the ICOSL 3′-UTR, disrupt the inhibitory effect of miR-24 on ISOSL expression. Consequently, SNP rs4819388 has an effective function in the progression of GC [56]. Therefore, targeting the ICOSL/ICOS pathway could be used to improve GC therapy.

## 4. B7-H1 (PD-L1) and B7-DC (PD-L2)

Programmed cell death-ligand 1 (PD-L1, also assigned as B7-H1 or CD274) and programmed cell death-ligand 2 (PD-L2, also assigned as B7-DC or CD273) are two ligands of programmed cell death 1 (PD-1, CD279) (Figure 1, Table 1) [57,58]. The PD-1 encoding gene is PDCD1 with five exons, while the PD-L1 encoding gene is CD274 with seven exons [59]. The amino acid sequence homology between PD-L1 and PD-L2 is approximately 40 percent [60,61]. The soluble PD-L1 (sPD-L1) is the other form of PD-L1, which is mostly found in sera of healthy people. It has also been discovered in various cell lines of cancer [59]. Both PD-L1 and PD-L2 consist of one IgV and one IgC domain. The PD-1 structure with two tyrosine base/288 amino acids includes a membrane-permeating domain, extracellular domain, and cytoplasmic tail at C terminal [62]. Phosphorylation of PD-1 is accomplished in immunoreceptor tyrosine-based inhibitory motif (ITIM) and immunoreceptor tyrosine-based switch motif (ITSM). Accordingly, after PD-1 phosphorylation, the TCR signal is regulated through Src homology 2 domain-containing phosphatase 1 (SHP1) and SHP2 [59]. The affinity of PD-L2 and PD-L1 is 3:1 to bind with their PD-1 receptor [63]. It has been demonstrated that PD-L1 binds to either PD-1 or B7-1 (CD80). The interaction of PD-L1/PD-L2 with PD-1 boosts tolerance of T-cells, induces an inhibitory effect on T-cell activation/proliferation, increases the conversion of T helper cells into Foxp3+ Treg cells, and prevents cytolysis of T cell in cancerous cells. Consequently, it causes cancer growth and suppresses the immune system [64]. The interaction of PD-L1 with B7-1 (CD80) leads to a reduction in the production of cytokine and proliferation of T lymphocytes [65]. Expression of PD-1 occurs on the macrophages, natural killer cells (NK cells), T helper cells, cytotoxic T cells, dendritic cells, monocytes, B cells, and mainly on activated T lymphocytes [66]. The expression of PD-L1 individually in inflammatory situations is accomplished by epithelial cells, dendritic cells, activated T lymphocytes, macrophages, and B cells [67]. Moreover, it is prominently expressed in several cancers, such as gastric, multiple myeloma, renal cell carcinoma, melanoma, etc. However, PD-L2 expresses chiefly on APCs such as non-hematopoietic tissues, myeloid dendritic cells, and macrophages [66]. The result of a study revealed that the PD-L1 expression is enhanced through signaling pathways such as PI3K/Akt/mTOR. Moreover, they proved that immunoresistance mediated by PD-L1 could be suppressed by PI3K kinase pathway inhibitors [68]. High expression of PD-L1 inhibits anti-tumor immunity and increases chemoresistance in human cancers. In this regard, Wu et al. revealed that PD-L1 overexpression enhanced the ERK pathway activation in tumor cells. PD-L1 expression is also essential for P38 MAPK activation. It is noteworthy that activation of the ERK/P38 MAPK pathway depends on the association between the catalytic subunit of a DNA-dependent serine/threonine-protein kinase and PD-L1. Thus, the reason for cancer chemoresistance in tumor cells can be due to the above signaling pathways’ activation [69]. Targeting the PD-1/PD-L1 pathway via antibodies against them is one of the remarkable treatment strategies in different types of cancer. Particularly, pembrolizumab, nivolumab, and pidilizumab can target PD-1 while atezolizumab, durvalumab, avelumab, and BMS-936559, which are anti-PD-L1 antibodies [70]. According to a study, the suppression of PD-1/PD-L1 expression induces the expression of miR-21 in GC. It is also indicated that the Treg cell number decreased while the Th17 cell number enhanced. Hence, the study showed that miR-21 is negatively controlled by the PD-1 [71]. Zhang and colleagues concluded that PD-L1 is overexpressed at high levels in GC patients [72]. Furthermore, it is proved that in GC, activation of the hedgehog (Hh) signaling increases the expression of PD-L1 and induces resistance in cancer immunotherapy. It is proposed that amelioration of therapeutics is possible through combination drug therapy with inhibition of immune checkpoints and Hh signaling in GC patients [73]. Geng et al. showed that PD-L1 was directly correlated with metastasis in lymph nodes, tumor depth of invasion, and Foxp3+ expression. They demonstrated that Foxp3+ and PD-L1 have a negative prognostic role in GC [74]. Moreover, Wang and colleagues demonstrated that PD-L1 could be overexpressed in GC due to somatic mutations, such as guanine allele to cytosine allele mutation at the 3′-untranslated region (3′-UTR) of the PD-L1. Moreover, miR-570 has a post-transcriptional regulatory and inhibitory role in PD-L1 expression. Mutation in PD-L1 3′-UTR disrupts the inhibitory role of miR-570 and decreases binding affinity to miR-570. Therefore, the level of miR-570 expression is reduced after overexpression of PD-L1 in GC [75]. Imai and colleagues showed that Interferon-γ (IFN-γ) induced the expression of membranous and intracellular PD-L1 in GC cells. Additionally, a noticeably high concentration of sPD-L1 was observed not only in the GC patient’s serum but also in the GC supernatant [76]. It has also been demonstrated that loss of heterozygosity (LOH) at the PTEN gene leading to PD-L1 represents a high level of expression in GC patients. GC with a mutation in the PIK3CA that causes PD-L1 is also considerably expressed [77,78]. Deng et al. indicated that expression of PD-L1 in GC considerably is associated with the expression of histone deacetylase 1 and 3; additionally, IFN-γ can induce expression of PD-L1 in this cancer. They showed that the suppression of histone deacetylase decreased IFN-γ-induced PD-L1 expression. Thus, it is suggested that targeting the PD-L1 expression via small molecular inhibitors of histone deacetylase could be useful in GC therapy [79]. It is revealed that PD-L2 is expressed significantly in a special type of GC known as Epstein–Barr virus (EBV)-associated GC (EBVaGC) [80]. Nakayama et al. revealed that expression of PD-L2 is detected in 28.4 percent of tumor cells related to patients with GC. PD-L2 expression is increased through IFN-γ in these patients. Furthermore, IL-4 induces the PD-L2 expression. Consequently, IFN-γ and IL-4, due to the PD-L2 upregulation, can promote GC progression [81]. Using multicolor flow cytometry, SAITO and colleagues proposed that elevated levels of PD-L1 expression were observed on both CD4+ and CD8+ T cells in GC tissue. Additionally, they presented that a high level of PD-1 expression on CD4+ and CD8+ T cells can induce immune system evasion in GC patients [82]. Chang and colleagues found that higher levels of CD8+ tumor-infiltrating lymphocytes (TIL) from patients with gastric adenocarcinoma can be associated with a poor prognosis value of PD-L1 expression [83]. Another study demonstrated that PD-L1 expression is detected in the immune stromal and tumor cells of GC. They showed that enhancing infiltration of CD8 T lymphocytes is associated not only with a high level of PD-L1 expression but also with poor OS and progression-free survival (PFS) in these patients [84]. As a result, according to the studies mentioned above, the levels of PD-L1 and PD-L2 (especially PD-L1) are overexpressed in GC patients.

## 5. B7-H3 (CD276)

B7-homolog 3 protein (B7-H3; also termed CD276 and B7 relative protein 2 (B7RP-2)) is recognized as a 45–66 kDa transmembrane glycoprotein (type I) in the B7 family immune checkpoints. B7-H3 with ten exons shares about 20–27% of identical amino acid sequences with other B7 family members. Initially, B7-H3 was defined in humans and later in mice. The B7-H3 gene is situated on chromosomes 15 and 9 in humans and mice, respectively [85,86]. The B7-H3 extracellular domain is composed of two similar pairs of the IgV and IgC domains (VCVC) in humans because of duplication of the exon, but in mice, it consists of one IgC and one IgV domain. Extracellular IgV–IgC domains are encoded through exons 4-7 of the B7-H3 gene [85]. The expression of B7-H3 mRNA is so broad that it is expressed on numerous cell types and tissues. Likewise, it is ubiquitously detected in both the nonlymphoid and lymphoid organs. However, protein expression is not only infrequent and at low levels but also significantly limited to cell types such as NK cells, B cells, T cells, monocytes, and activated DCs [87]. B7-H3 can be expressed considerably in several cancers such as breast, stomach, colorectum, lung, etc. [88]. Analysis of the association between miR-29 and B7-H3 expression revealed that the low expression rate of miR-29 induces overexpression of B7-H3 in tumor tissue. MiR-29 not only has a modulatory role in the B7-H3 expression level in cultured cell lines but also targets B7-H3 mRNA [89]. It was demonstrated that B7-H3 has a co-stimulatory role i CD4 and CD8 T cell proliferation in the presence of an anti-CD3 antibody.B7-H3 also enhances cellular immune responses and IFN-γ generation in the presence of TCR signaling [86]. In comparison to the co-stimulatory role of B7-H3, most studies have generally shown that B7-H3 predominantly has a negative regulating role on T lymphocytes in humans and murine [90,91]. Moreover, B7-H3 inhibits the proliferation of T helper cells and cytotoxic T cells and decreases the production of IL-2 and IFNγ [90]. It is suggested that B7-H3 expression suppresses T cell activity by inhibiting or moderating transcription factors such as a nuclear factor of activated T cells (NFAT), AP-1, and NF-κβ [92]. Likewise, Veenstra et al. presented acceptable proof that B7-H3 is considered a co-inhibitory molecule and reduces activation of T cells [93]. It was also shown that B7-H3 suppresses the function of NK cells and induces mineralization in bone tissues and differentiation of osteoblast [94,95]. Collectively, B7-H3 can function as a co-stimulatory and co-inhibitory molecule; the difference in its role could be related to the existence of different immune cells/cytokines and multiple unidentified binding partners (Figure 1, Table 1) [6]. Triggering receptor expressed on myeloid cells (TREM)-like transcript 2 (TLT-2, TREML2) has been reported as a murine B7-H3 receptor which is generally accepted by most studies [96]. Some studies did not find proof of the presence of an interaction between B7-H3 and TLT-2. They proved that TLT2 is not considered as a corresponding receptor for B7-H3 [97,98]. To date, the putative receptor as a binding partner for B7-H3 remains uncertain [99]. In this regard, the result of a study revealed that an anti-B7-H3 monoclonal antibody such as humanized 8H9 could suppress the inhibitory effect of B7-H3 in humans through binding to the FG loop [100]. Another monoclonal antibody (mAb) against B7-H3 is enoblituzumab (MGA271), which inhibits the development of tumors [101]. Wu and co-workers demonstrated that the B7-H3 expression is detected in 58.8% of GC samples and in all gastric adenoma samples. They reported that patients with GC who have low levels of B7-H3 expression have a survival rate that is half that of patients with high levels of B7-H3 expression. B7-H3 expression is associated not only with survival in GC patients but also with tumor infiltration depth. A low level of B7-H3 expression causes the tumor to escape the immune system. Therefore, the level of B7-H3 expression is a good indicator for the prognosis and diagnosis of GC patients [102]. Furthermore, it was indicated that B7-H3 is considerably expressed in GC patients in comparison with the normal gastric group. They showed that in an orthotopic transplantation GC model, suppression of B7-H3 via RNA interference reduces metastasis such as invasiveness and migration in vitro and in vivo. Thus, B7-H3 might have a main function in the regulation of GC metastasis [103]. It has also been demonstrated that enhanced expression of B7-H3 is associated with the CXCR4 expression and tumor infiltration depth in GC patients. Additionally, the ability of cell migration and invasion in GC is reduced by suppressing B7-H3 by shRNA and decreasing the activity of signaling pathways such as Jak2/Stat3, ERK, and AKT in the GC cells [104]. Furthermore, overexpression of B7-H3 and activated phenotype of CD54+ has been detected on neutrophils in GC tumors, which is directly related to the detection of granulocyte-macrophage colony-stimulating factor (GM-CSF). Therefore, GM-CSF-activated neutrophils express B7-H3 that resulted in tumor aggressiveness in human GC by activation of the JAK-STAT3 signaling pathway [105]. Recently, Zhan and colleagues indicated that B7-H3 expression is remarkably observed in stromal and tumor cells of gastric adenocarcinomas (GACs); also, a high level of stromal B7-H3 expression is correlated with high expression of alpha-smooth muscle actin protein in these patients. They showed that inhibition of B7-H3 via small interfering RNA leads to reducing the potency of invasion and migration in cancer-associated fibroblasts (CAFs) and suppression of cytokine secretion. Therefore, elevated expression of B7-H3 might serve as a poor prognosis indicator for patients with GACs [106]. It is demonstrated that expression of B7-H3 can induce radiotherapy resistance of GC cells via modulating DNA double-strand break repair, cell cycle, and apoptosis. Moreover, cell autophagy baseline levels are regulated by B7-H3 in GC tissue, indicating that radioresistance in GC cells is caused by the expression of B7-H3 via regulation of cell autophagy baseline levels [107]. By immunohistochemistry and analysis of the digital image, Ulase et al. demonstrated that B7-H3 is expressed chiefly in the stromal compartment of patients with GC, and CD8+ T lymphocytes are inhibited in tumors with excessive expression of B7-H3. As a result, they reported that B7-H3, as a considerable target of GC immunotherapy, can regulate cell-mediated immunity responses in patients of Western origin [108]. Accordingly, the findings of various studies demonstrated that expression of B7-H3 is increased in GC patients.

## 6. B7-H4 (B7x)

B7 homolog 4 (B7-H4), also named-B7x and B7S1, belongs to the immune checkpoints of the B7 family. B7-H4 is a transmembrane protein, and the DNA sequence homology of this immune checkpoint with other B7 family members resulted in its identification in 2003. The encoding gene related to B7-H4 is VTCN1, which encodes 282 and 283 amino acids in humans and murine, respectively [109,110]. Structurally, B7-H4 consists of the intracellular domain, a hydrophobic transmembrane domain, and an extracellular domain [111]. B7-H4 consists of one IgV and one IgC domain. It is situated on chromosomes 3 and 1 in murine and humans, respectively [112]. B7-H4 is an inhibitory member of immunoregulatory molecules that do not bind to the CTLA4/CD28, ICOS, and PD-1 (Figure 1, Table 1) [110]. To date, a specific receptor for binding to B7-H4 has not been recognized; however, B and T lymphocyte attenuator (BTLA) was suggested as a B7-H4 receptor that binds indirectly, while further experiments have not accepted this proffer [113,114]. B7-H4 mRNA can be expressed in human renal cell carcinoma, GC, and ovarian cancer. B7-H4 is also discovered in the peripheral blood (PB) and tumor microenvironment (TME) [115]. It is proved that B7-H4 has low expression in normal tissues [112]. The flow cytometric analysis demonstrated that B7-H4 does not express on the surface of dendritic cells, monocytes, and B/T cells in humans. However, the expression of B7-H4 in vitro could be moderately increased on such cells following stimulation with ionomycin, IFN-γ, phorbol 12-myristate 13-acetate, phytohemagglutinin, and lipopolysaccharides (LPS) [110]. Some studies have indicated that modification of B7-H4 expression depends on cytokines. For example, IL-10 and IL-6 increase the expression of B7-H4; however, IL-4 and DC-differentiation cytokines reduce their expression [116,117]. In this regard, Kryczek and colleagues have suggested that Treg cells stimulate the macrophages to generate IL10 and IL6. Thus, B7-H4 can be upregulated and inhibits T cell proliferation [118]. Contradictory results have been presented regarding the B7-H4 antitumor immunity by using various samples of mouse tumors and diversity in B7-H4 expressing cell types. In 2015, a study indicated that B7-H4 is necessary for antitumor immune responses and inhibits the development of tumors [119], while most studies have proved that B7-H4 has a negative modulatory role in T cell antitumor immune responses [112]. Previous studies have proved that recombinant anti-B7-H4 antibodies are a possible strategy for improving anti-tumor immunity and inducing T-cell response [120,121]. Recently, Zhou et al. have revealed that B7-H4 expression in tumor cells of mouse models not only decreased the production of IFN-γ but also negatively regulated CD8 tumor-specific T cell cytotoxicity, expansion, and activation. Consequently, B7-H4 overexpression leads to tumor growth and weakens tumor immunity in the mouse tumor model [122]. Arigami and co-workers proved that a high level of B7-H4 mRNA expression was detected in the peripheral blood lymphocytes (PBLs) of GC patients but not in normal PBLs from healthy volunteers. They realized that B7-H4 expression is a helpful factor for predicting GC progression and prognosis [115]. In addition, Jiang and colleagues demonstrated that the B7-H4 expression in GC patients was remarkably higher than tissues with a gastric polyp and epithelial of normal gastritis. They also indicated that B7-H4 induces metastasis of nodal and invasion of tumors in GC and revealed that B7-H4 has a prognostic marker value for GC [123]. It is also indicated that poor survival of GC patients in the advanced stage is the result of the development of neutrophils in the environment of GC. Moreover, GM-CSF stimulates neutrophils and increases the expression of B7-H4 via activation of JAK/STAT signaling. The result of this study revealed a new modulating mechanism of B7-H4 expression on neutrophils that can activate tumors in GC, proposing that efficient suppression of these new GM-CSF-B7-H4 pathways could be an appropriate strategy in GC therapy [124]. In another study, Geng et al. indicated that B7-H4 was directly associated with the expression of Foxp3+ and lymph node metastasis. They presented that low Foxp3+ expression is associated with better survival of GC patients [74]. Furthermore, the result of a meta-analysis study in 2016 showed that blood B7-H4 is an indicator of poor prognosis in GC patients [125]. It was proved that the inhibition of B7-H4 via siRNA not only reduces carcinogenesis of the MGC-803 human GC cell line but also increases apoptosis [126]. It has been revealed that the number of tumor-infiltrating lymphocytes (TIL), as indicators of good outcome of survival, is reduced in GC patients with overexpression of B7-H4 [127]. It is also indicated that neoadjuvant chemotherapy (NACT) of GC patients induces the presence of high CD4+ and CD8+ TIL levels and reduces the expression of B7-H4. Thus, downregulation of B7-H4 might be considered as a proper biomarker for response prediction of NACT in GC patients [128]. Consequently, the expression of B7-H4 as a prognostic factor can be considered an attractive target for GC therapy.

## 7. B7-H5 (VISTA)

The presence of another molecule of the B7/CD28 superfamily, V-domain immunoglobulin suppressor of T cell activation (VISTA; also called B7-H5, PD-1 homolog (PD-1H), DD1α, Dies1, SISP1, and Gi24), has been recently reported, which can be a potential target in immuno-oncology [129,130]. VISTA is a well-defined type I immunoglobulin membrane protein with 279 amino acids, including cytoplasmic tail (96 amino acid residues), transmembrane domain (TMD) (21 amino acid residues), and extracellular domain (162 amino acid residues). The extracellular domain related to VISTA with homology to PD-1 and IgV-like domain of PD-L1 contains only one IgV domain [129,131]. The encoding gene of VISTA is Vsir, which is not only situated on chromosome 10 at a distant position from all members of the immunoglobulin superfamily but also is located within the intron of the CDH23 gene. Sequencing analysis of the VISTA gene disclosed that VISTA remarkably has a high level of conservation among members of the B7 family and represents 76 percent homology between humans and mice [132]. The expression of VISTA primarily but not particularly was detected in tissues that have considerable numbers of leukocytes and hematopoietic tissues. VISTA is overexpressed in myeloid cells and is associated with a reduced expression on CD4+ and CD8+ T lymphocytes. VISTA can be expressed at a high level on forkhead box protein 3 (FoxP3) + T regulatory cells and naive CD4+ T lymphocytes. VISTA represents low levels of expression on CD8+ T lymphocytes and NK (CD56hi) cells. Although there was no detectable VISTA expression on B cells, its expression has been reported considerably on plasma cells [133,134,135]. Previous studies revealed that VISTA could be expressed in human cancerous tissues such as colorectal cancer, GC, non-small cell lung cancer, pancreatic, ovarian cancer, prostate cancer, hepatocellular carcinoma, acute myeloid leukemia, and metastatic melanoma [135]. The expression of VISTA is upregulated through the enhancement of transcription factors such as hypoxia-inducible factor 1-alpha (HIF-1α) and p53 [136,137]. Deng et al., using chromatin immunoprecipitation, demonstrated that HIF-1α could induce VISTA on myeloid cells in the tumor microenvironment (TME) and hypoxic situations [137]. VISTA participates in the controlling of immune system reactions due to possessing the main homeostatic role. This function of VISTA is the result of a specific and wide pattern of its expression. Therefore, there is discrimination between VISTA and other immunomodulatory receptors [138]. It is proved that VISTA acts as a co-inhibitory ligand on APCs, while it acts as a co-inhibitory receptor when expressed on CD4+ T lymphocytes. The proliferation and production of IFN-γ and IL-2 are suppressed due to the VISTA expression on APCs. The expression of VISTA on CD4+ T lymphocytes resulted in the suppression of T cells [133,139,140]. In 2019, Wang et al. indicated that VISTA could act as a receptor. They demonstrated that the V-Set and Immunoglobulin domain containing 3 (VSIG-3) could be introduced as a new ligand partner for the VISTA. To date, the biological importance of VSIG-3/VISTA engagement is not identified. The interaction of VSIG-3 with VISTA as ligand receptors resulted in sending the co-inhibitory signals for the suppression of cytokine/chemokine production and the function of T lymphocytes, in vitro [141]. Another study in 2019 demonstrated that VISTA is a ligand and P-selectin glycoprotein ligand-1(PSGL-1) is a receptor for VISTA. The researchers presented that engagement of PSGL-1 with VISTA takes place in acidic environments [142]. Collectively, VISTA is considered both as a ligand and receptor and has a co-inhibitory function (Figure 1, Table 1) [139]. In mouse tumor models, LeMercier et al. presented that using monoclonal antibodies against VISTA resulted in reduced an inhibitory function of VISTA and the number of tumor-associated FoxP3+ CD4+ Tregs. On the other hand, blocking the VISTA pathway induced the activity of intratumoral T lymphocytes. Hence, anti-VISTA antibodies increase the immune response to tumors, production of IFNγ/TNFα, and expression of CD44. Consequently, VISTA represents a promising novel target for immunotherapy of cancer, either on its own or in combination with another immune checkpoint blockade [133]. A recent study indicated that combination therapy of anti-VISTA and anti-PD-1 antibodies is efficient in mouse tumor models and may be an optimal option for treating cancers [143]. The outcome of combination immunotherapy using anti-VISTA plus anti-PD-L1 or anti-PD-1 antibodies increased TNFα, IFNγ, and serine protease such as granzyme B (GrzB) in CD8+ T lymphocytes and decreased the tumor growth [129]. Hu and co-workers revealed that VISTA is overexpressed not only in the nucleus but also in the cytoplasm of GC patients’ tissues. They showed an inverse correlation between OS rate and cytoplasmic VISTA expression at a high level in GC patients. In contrast, the five-year OS rate in GC patients with high nucleus VISTA expression is identical with GC patients, who have a low level of nucleus VISTA expression [144]. Oliveira et al. revealed that the expression of VISTA is reduced in GC cell lines compared to normal gastric tissue through the methylation of promoter-like methylation of CpG sites at the 5′ end of the Vsir gene and/or overexpression of miR-125a-5p [145]. Boger and colleagues, using immunohistochemistry, demonstrated that the VISTA expression in immune and tumor cells was observed in 83.6 and 8.8 percent of patients with GCs, respectively. The VISTA expression of immune cells remarkably is augmented from tumor grade pT1 to pT2, but reduced from pT2 to pT3. A change in VISTA expression was detected in tumors during the progression of GC. Moreover, researchers presented that the expression of VISTA in GC is correlated not only with PD-L1 expression but also with Epstein–Barr virus infection, tumor localization, Lauren phenotype, and KRAS-and PIK3CA-mutational status, while no correlation exists between the expression of VISTA with tumor stage, distant metastasis, and lymph node metastasis. Consequently, the predictive role of VISTA expression as a biomarker has not been assessed in patients with GC, and more research is required in this regard [146].

## 8. B7-H6 (NCR3LG1)

The B7 homolog 6 (B7-H6), also named NCR3LG1, belongs to the immune checkpoints of the B7 family and functions as an endogenous/co-stimulatory ligand. In 2009, Brandt’s study using techniques such as mass spectrometry and bioinformatics resulted in detecting B7-H6 as a type 1 transmembrane protein [147]. The corresponding receptor of B7-H6 is NKp30, which is a natural killer (NK) cell-activating receptor (Figure 1, Table 1) [148]. It is reported that the extracellular region of NKp30 contains one IgV-like domain while the extracellular region of B7-H6 comprises IgV/IgC-like domains. B7-H6 intracytoplasmic domain includes SH2/SH3-binding motif and Immunoreceptor tyrosine-based inhibitory motif (ITIM) [149]. It is revealed that the gene sequence of B7-H6 is homologous to PD-L1 and B7-H3 [147]. On tumor cells, the binding of B7-H6 to NKp30, which is located on NK cells, induces the function of NK cells to identify tumors through the production of interferon-γ. Therefore, B7-H6 acts as an alarm system to alert innate immune responses [148]. Moreover, B7-H6 mRNA and protein expression are seen in different kinds of tumor cell lines, B/T cell lymphoma, and leukemia, but are absent in healthy peripheral blood mononuclear cells (PBMCs) or normal human tissue. Th expression of B7-H6 at high levels is detected in several types of cancer including breast cancer, lung cancer, melanoma, liver cancer, ovarian cancer, renal cell carcinoma, hepatocellular carcinoma, cervical carcinomas, brain cancer, and GC [147]. Matta and colleagues showed that B7-H6 is chiefly expressed at the neutrophils and pro-inflammatory CD14+ CD16+ monocytes surface. It is proved that activated neutrophils and monocytes can release soluble B7-H6. they also realized that IL-1β and tumor necrosis factor-α (TNF-α), as well as toll-like receptor (TLR) ligands, can induce expression of B7-H6 [150]. Both immunological and non-immunological functions of B7-H6 are involved in tumorigenesis. It has been demonstrated that B7-H6 has an anti-apoptotic role and the initiation of caspase cascades is inhibited via B7-H6. Therefore, B7-H6 can increase tumorigenesis in tumor cells through the activation of a transcription factor such as the STAT3 signaling pathway [151]. Moreover, activation of apoptosis inhibitors through the STAT3 pathway causes that B7-H6 induces proliferation in tumor cells [152,153]. Additionally, it is revealed that the immunological role of B7-H6-induced tumorigenesis by IFN-γ/TNF-α secretion and control of bi-specific T-cell engagers (BiTEs) triggers T cell cytotoxicity [154]. As B7-H6 is expressed in various types of cancer, it is a good candidate for targeted therapy, and using specific monoclonal antibodies against B7-H6 has been considered as a promising treatment of tumors [155]. It is revealed that mouse single-chain variable fragment (scFv)-based chimeric antigen receptors (CARs) can increase the anti-tumor function of T cells by targeting B7-H6 [156]. Chen et al., using immunohistochemistry, showed that the prognostic role of B7-H6 was not observed in GC patients. They realized that the B7-H6 expression is the same between gastric tumors and non-tumor adjacent tissue. No relevance was seen between the expression of B7-H6 and clinical features such as sex, age, distant metastasis, histological classification, lymph node metastasis, tumor size, and TNM stage. However, they demonstrated that a high degree of tumor differentiation is considerably associated with the positive expression of B7-H6 in gastric tumors [157]. On the other hand, in contrast to the above result regarding the predictive role of B7-H6 in patients with GC, it is demonstrated that a considerably high level of B7-H6 expression is a good predictor of OS for GC patients [158]. Hence, more research is required to recognize the prognostic value of B7-H6 in GC.

## 9. B7-H7 (HHLA2)

HHLA2 is an abbreviation form of the human endogenous retro virus-H long terminal repeat associating-2, also named B7-H7 and B7y [159]. Screening in sequences of expressed genes (expressed sequence tags; ESTs) resulted in HHLA2 identification in 1999 [160]. Afterward, HHLA2 was detected as a member of the B7 ligand family. HHLA2 shares about 23–33% of similar amino acid sequences with other B7 family members. HHLA2 contains two Ig variable-like (IgV) domains and one Ig constant-like (IgC) domain (IgV-IgC-IgV) [161,162]. According to a study by Zhu and colleagues, B7-H7 was before named B7-H5. However, at present, B7-H7 and B7-H5 are referred to as HHLA2 and PD-1H, respectively [161,163]. HHLA2 is found in humans but not in mice [164]. Zhu et al., through receptor array screening, indicated that CD28 homolog (CD28H) as a receptor could interact with HHLA2 on APCs [163]. Then, Janakiram et al. demonstrated that transmembrane and immunoglobulin domain-containing 2 (TMIGD2) could bind to humans HHLA2. TMIGD2 as a receptor is situated on chromosome 19q13.3 [165]. Sequencing analysis discloses that TMIGD2 and CD28H are similar molecules. Consequently, TMIGD2/CD28H are now considered as HHLA2 receptors [159]. This receptor–ligand interaction takes place on the different subsets of main immune cells such as CD4/CD8 T lymphocytes, and APCs (dendritic cells, B cells, monocytes) [161]. Previously, Janakiram et al., using immunohistochemistry, showed that the HHLA2 expression was seen not only in the epithelium of the breast, gallbladder, gut, and kidney, but also in trophoblastic cells of the placenta [159]. In the immune system of humans, HHLA2 represents high levels of expression on macrophages and monocytes, while there is no detectable HHLA2 expression on resting B or T cells and immature dendritic cells (DCs). Expression of HHLA2 at high levels also is detected in various types of cancer. In addition, expression of HHLA2 is induced on B lymphocytes and increased on the monocytes, and mature DCs subsequent stimulation with inflammatory signals such as poly I: C, IFN-γ, and lipopolysaccharide (LPS) [161,163,164]. Zhu et al. indicated that HHLA2 has a co-stimulatory role when it binds to the CD28H receptor on APCs. Consequently, the proliferation of T lymphocytes and cytokine production is increased through the AKT pathway [163]. In contrast to the co-stimulatory role of HHLA2, it has a co-inhibitory effect on the responses of T lymphocytes. The proliferation of CD4+ and CD8+ T lymphocytes is suppressed via HHLA2 in the existence of TCR signaling. The production of cytokine-like IFNγ, TNFα, IL-5, IL-10, IL-13, IL-17a, and IL-22 is considerably decreased by incubating T lymphocytes with HHLA2 [161]. Collectively, HHLA2, like B7-H3, has co-stimulatory also co-inhibitory function on T lymphocytes in various situations (Figure 1, Table 1) [161,163]. The broad expression pattern of HHLA2 showed that HHLA2 has a role in developing tumors by inhibiting anti-tumor immune responses. Interestingly, the HHLA2 pathway is considered an appropriate target for cancer immunotherapy using ICIs and treatment with antibody-drug conjugates (ADCs) [159]. Targeting HHLA2 resulted in increased immune responses against tumors and the angiogenesis of tumors being suppressed [165]. Shimonosono and colleagues, using immunohistochemistry, examined the level of HHLA2 expression in the blood samples of GC patients and healthy people. It has been shown that normal epithelial cells expressed the highest level of HHLA2 compared with tumor cells. The researchers presented that the level of HHLA2 mRNA expression in blood samples of patients with GC is considerably lower than in peripheral blood mononuclear cell (PBMC) samples of healthy people. They also found that expression of HHLA2 at a low level resulted in tumor aggressiveness, adverse prognosis, as well as a lower five-year survival rate in GC patients. A low level of HHLA2 mRNA expression correlates with disease stage, distant metastasis, and tumor invasion depth in the blood of patients with GC. Consequently, the evaluation of HHLA2 expression in blood samples might be used not only to distinguish healthy people from GC patients but also to prognosticate the aggressiveness of GC [166]. On the other hand, in contrast to the above results regarding the prognosis of patients with GC, Wei et al., using two techniques such as immunohistochemistry and qRT-PCR, demonstrated that both the protein and mRNA expression HHLA2 are increased in the tissue of GC in comparison with normal gastric tissue. They concluded that overexpression of HHLA2 is correlated with poor OS outcomes. Therefore, patients with GC have lower OS due to higher expression levels of HHLA2 compared to those of patients with low expression of HHLA2. The HHLA2 expression at high levels is correlated with the presence of distant metastasis, metastasis in lymph nodes, deep tumor invasion, and advanced clinical-stage, while no considerable correlation exists between the expression of HHLA2 with the location of the tumor, age, gender, the histologic tumor grade, and Lauren’s classification in GC patients. Consequently, overexpression of HHLA2 is considered not only as a remarkable risk factor for the malignant status of GC tissue but also as a biomarker of undesirable prognostic for OS in GC patients [167]. It is noteworthy that the difference between Shimonosono et al.’s and Wei et al.’s results is most likely owing to the weakly negative correlation between protein expression levels of HHLA2 in primary tumor tissues and mRNA expression levels of HHLA2 in samples of blood. Generally, the prognostic value of HHLA2 in GC patients needs to be investigated and approved by further studies.

## 10. Ig-like Domain-Containing Receptor 2 (ILDR2)

Ig-like domain-containing receptor 2 (ILDR2) has been newly identified as a B7-like ligand (Figure 1, Table 1) [168]. The encoding gene of ILDR2 is C1orf32, located on Chr1q23–25 in humans [169]. Human ILDR2 mRNA can encode type I membrane protein with 639 amino acids. This protein contains a signal peptide, which exists in N-terminus, the intracellular tail (433 amino acid residues), transmembrane domain (20 amino acid residues), and Ig V-set/-type (IgV) domain (167 amino acid residues) [168]. ILDR1 and ILDR3 (lipolysis-stimulated receptor) are known as the two paralogs of ILDR2 [170]. The human ILDR2 represents 94 percent homology with the murine ortholog. The amino acid sequence homology between ILDR2 and other members of the B7 family is approximately 24–36 percent. Protein expression of ILDR2 in humans has been observed on CD56+ lymphocytes, human monocyte-derived from macrophages, and CD16+ monocyte subsets. Expression of ILDR2 mRNA at high levels is detected in the brain and ovaries while it is low in the intestine, heart, and kidney tissues. Moreover, the mouse ILDR2 mRNA expression pattern is similar to the pattern of human ILDR2 mRNA expression [168]. Recombinant ILDR2-Fc fusion protein, without increasing apoptosis of T cells, leads to cell division and inhibits the activation of early TCR signaling. The agonistic activity of ILDR2-Fc on inhibitory receptors suppresses the CD4 and CD8 T lymphocytes activation using monoclonal antibodies against CD3 and CD28 in mice and humans [168]. Despite the inhibitory effect of ILDR2 in T lymphocytes activation, Podojil and colleagues previously showed that ILDR2 has a vital role in the induction of antigen-specific immune tolerance by increased activity of Treg cells and its significant role was revealed in both regulating and modulating immune homeostasis [171]. This immunomodulatory activity related to ILDR2 and its effect on antigen-specific immunological tolerance has been demonstrated in various autoimmune disease models such as mouse models in rheumatoid arthritis (RA), type I diabetes, and multiple sclerosis (MS) [168,171]. Recently, the efficacy of the BAY 1905254 as a human/mouse cross-reactive hIgG2 monoclonal antibody against ILDR2 was suggested by Huetter and colleagues. They presented that the antibody targeting ILDR2, BAY 1905254, enhances activation of T lymphocytes and induces responses of antigen-specific T lymphocytes in vivo via inhibiting the immunosuppressive role of ILDR2. Consequently, BAY 1905254 can induce anti-tumor immune responses in vivo, as monotherapy or in combination with docetaxel and anti-PD-L1 in various syngeneic tumor models [172]. To date, no studies have reported on the role of ILDR2 in pathological states and the clinical significance of ILDR2 expression in GC. Collectively, ILDR2 plays a possible shared role in the progress of both cancer and autoimmune diseases. Therefore, targeting ILDR2 via BAY 1905254 is effective for the immunotherapy of cancer [168].

## 11. Treatment of Gastric Cancer Base on B7 Family Inhibition

**Figure 2:**
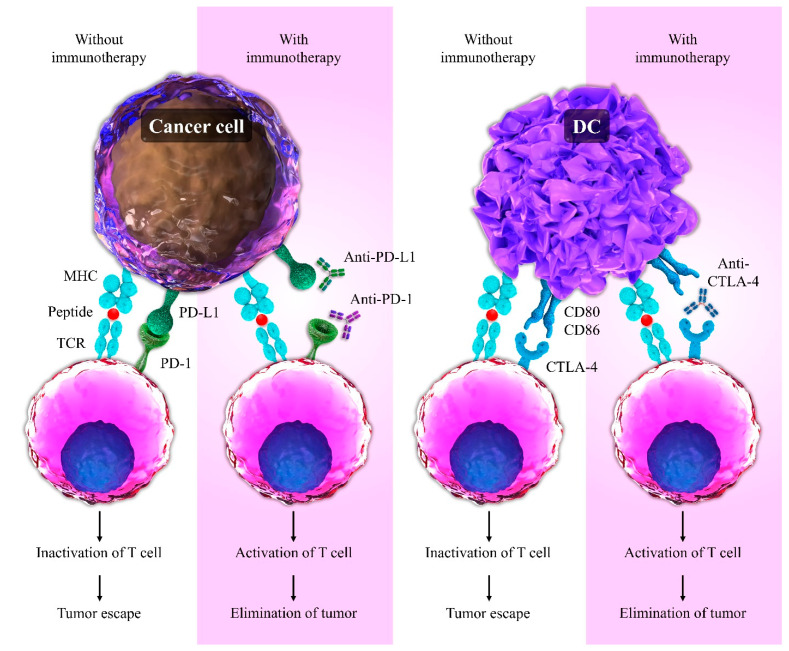
Comparison of the use of immunotherapy and non-use of this method in T cell response. Interaction of PD-L1 with PD-1 and CD80/CD86 with CTLA-4 leads to the inactivation of T lymphocytes, and tumors can escape from the immune system. In contrast, immunotherapy using CTLA-4 and PD-1-blocking antibodies causes the elimination of tumors by promoting increased T cell activation.

Different options are considered for the treatment of GC. Immunotherapy via ICIs is a novel approach for the management of GC [4]. ICIs using monoclonal antibodies have recently gained considerable attention in the treatment of cancer (Figure 2, Table 2). Ipilimumab (fully human IgG1 monoclonal antibody), as a type of anti-cancer drug, targets CTLA-4. In 2011, for the first time, the FDA (U.S. Food and Drug Administration) approved ipilimumab as a second-line setting for the treatment of metastatic melanoma due to its promising efficacy in this cancer [173]. CTLA-4 and PD-1-blocking antibodies were demonstrated to block signaling pathways such as PI3K/AKT, MAPK, and β-catenin in GC cells. These neutralizing antibodies considerably not only suppressed metastasis and epithelial–mesenchymal transition (EMT) in MGC-803 and MKN-45 cells but also increased the levels of apoptosis. Therefore, combination therapy using CTLA-4 and PD-1-blocking antibodies may improve favorable outcomes in GC patients [174]. Another study was conducted by Ralph et al. regarding inhibition of CTLA-4 using tremelimumab as a monoclonal antibody in esophageal adenocarcinoma and metastatic GC patients. Their results show that tremelimumab has an effect in combination with different kinds of immunotherapy [175]. It has been demonstrated that immunotherapy drugs such as nivolumab against PD-1, as monotherapy or in combination with ipilimumab against CTLA-4, provide clinical benefit responses (CBR) in phase I/II CheckMate-032 trial. According to this trial, the Overall Response Rate (ORR) in patients who received nivolumab 3 mg/kg Q2W (N3) was 12%. Likewise, the ORR was 24% and 8% in groups of patients who received nivolumab 1 mg/kg + ipilimumab 3 mg/kg Q3W (N1 + I3) and nivolumab 3 mg/kg + ipilimumab 1 mg/kg Q3W (N3 + I1), respectively [176]. The phase Ib KEYNOTE-012 trial showed that using pembrolizumab can induce anti-tumor immune responses in GC patients with positive PD-L1 expression [177]. Nivolumab, as a checkpoint inhibitor drug that targets PD-1, could improve OS in patients with advanced GC that before received chemotherapy at least two times. The approval is based on the ATTRACTION-02 trial. A phase III trial showed that median survival in the groups that received nivolumab was 5.26 months, while for the groups that received the placebo, median survival was 4.14 months. These results suggest that in prior lines of therapy, nivolumab could be useful as a novel treatment method and care standard in patients with advanced GC that was previously not responsive to chemotherapy [178]. It is noteworthy to report that the KEYNOTE-062 phase III is one of the key trials in the first-line therapy of advanced GC. This trial indicated that 69 percent of GC patients have a PD-L1 combined positive score (CPS) ≥ 10, and GC patients with CPS ≥ 10 who received pembrolizumab have prolonged OS in comparison to those patients who received chemotherapy. However, in patients with CPS ≥ 1, the OS is 11.1 and 10.6 months in groups that received only chemotherapy and pembrolizumab, respectively. This study revealed that in first-line therapy, pembrolizumab compared to chemotherapy has clinically significant effects on the OS of GC patients whose tumors have a CPS score ≥ of 10 [179]. It has been demonstrated that in the phase Ib JAVELIN solid tumor trial, avelumab as first-line maintenance (1 L mn) or second-line (2 L) therapy presented a safety profile with less toxicity in patients with GC. The anti-tumor activity of avelumab as an anti-PD-L1 monoclonal antibody was detected in GC patients. It is important to note that more studies are essential for supporting this treatment strategy because of the result of this trial was for a small percentage of GC patients [180]. The phase III KEYNOTE-061 trial was performed in second-line therapy for GC patients with high microsatellite instability (MSI-H) and deficiency of mismatch repair that had disease development due to the use of fluoropyrimidine and platinum as first-line therapy. In this trial, the OS was not reached considerably with pembrolizumab because the OS in patients who were receiving pembrolizumab was 9.1 months while in the chemotherapy group, OS was 8.3 months. However, the use of pembrolizumab over paclitaxel is useful in GC patients with MSI-H [181]. The phase II Clinical KEYNOTE-059 trial approved the use of pembrolizumab in third-line setting for PD-L1 expressing GC who previously received ≥2 lines of therapy such as platinum and fluoropyrimidine. In this trial, ORR was 11.6% and 15.5% in all eligible patients and in patients who tested positive for PD-L1, respectively [182]. Another result from the phase III, randomized JAVELIN Gastric 300 trial showed that avelumab as a third-line monotherapy has negative consequences regarding ORR, OS, and PFS in advanced GC patients, while patients who received chemotherapy drugs such as paclitaxel and irinotecan have higher PFS, OS, and ORR. On the other hand, the antitumor activity appeared similar in patients who received chemotherapy or avelumab. However, the safety profile of avelumab is manageable in comparison to chemotherapy [183]. Moreover, the result of a meta-analysis study in 2019 revealed that in GC patients, using anti-PD-1/PD-L1 therapy with a low risk of side effects is more efficient in comparison to anti-CTLA4 therapy. PD-1/ PD-L1 blockade therapy showed better efficacy in GC patients with positive PD-L1 expression, who were Epstein–Barr virus-positive (EBV+), had tumors with high mutation burden, and had high microsatellite instability (MSI-H) [184]. Due to the existence of an association between VISTA and PD-L1 expression in patients with GC, the combination of VISTA and PD-L1 blocking antibodies might be practicable in the treatment of GC patients, considering that it was effective in mouse tumor models [146]. Since VSIG-3 has been discovered as a ligand for the VISTA, it is suggested that targeting the VSIG-3/VISTA pathway via blocking antibodies provides a new approach for the immunotherapy of GC [144]. Targeting B7 family members with blocking antibodies may limit treatment due to side effects in some patients with cancer. Immune-related adverse events (irAEs) such as pneumonitis, endocrinopathies, hepatitis, colitis, and rash are observed. Additionally, it is noteworthy that the occurrence of this side effect is higher in cases where the ipilimumab antibody is used in comparison with cases that are treated with pembrolizumab and nivolumab. Combination therapy of ipilimumab and nivolumab is associated with the prevalence of irAEs. Moreover, skin disorders such as neutrophilic dermatosis are reported as an unfavorable effect of using blocking antibodies in management of cancers. In general, targeting the immune checkpoint proteins using antibodies can be effective in boosting the immune system ability to fight cancer such as GC; thus, the treatment and management of irAEs is necessary so that the use of infliximab or corticosteroids as immunosuppression leads to treatment of irAEs [185,186].

## 12. Conclusions

GC is one of the most prevalent kinds of cancer and heterogeneous malignancy. A combination of particular genetic alterations, as well as environmental risk factors, account for GC. Delays in the diagnosis and the consequently high incidence/mortality of this cancer are significant reasons GC is such a challenging malignancy. Surgery, chemotherapy, and sometimes radiation therapy is most often used to treat GC patients. However, due to the low survival rates of these patients after the use of common treatments, it is demonstrated that immunotherapy has a prominent function in cancer therapy. One of the methods related to immunotherapy is using ICIs targeting the inhibitory B7 family members, which delivered promising results for patients with GC. Several members of the B7 family have been recognized as having considerable functions in regulating and directing T cell fate through their interactions with corresponding receptors. The expression of members of this family has clinical significance in GC. Evaluation of the B7 family members’ expression can be a valuable prognostic factor for early diagnosis and treatment in GC patients. For the management of GC, several clinical trials of different phases have been conducted to study the effect of using blocking antibodies against members of the B7 family. Mainly, targeting the PD-1/PD-L1 axis by neutralizing antibodies such as pembrolizumab and nivolumab has been appearing as a new approach in the management of advanced GC. Despite the fact that ICIs in different clinical trials are considered as a novel method in the advanced GC treatment, emerging new approaches are required to confirm the most efficient ICIs in GC, either as monotherapy or through combining ICIs with vaccines, radiotherapy, and agents with immunomodulatory characteristics [198]. Moreover, combination therapy can overcome the challenges of shorter-lasting cytotoxic chemotherapy responses and the delayed response of immune checkpoint inhibitors. Consequently, ICIs might pave the acceptable standard way of care for the treatment of GC patients.

## Figures and Tables

**Table 1:** Classification of the B7 family ligands and their receptors.

B7 Family Ligands	Alias Name	Corresponding Receptor(s)	Function
B7-1	CD80	CD28/CTLA-4	Co-stimulatory/Co-inhibitory
B7-2	CD86	CD28/CTLA-4	Co-stimulatory/Co-inhibitory
B7-H1	PD-L1/CD274	PD-1(CD279)/CD80	Co-inhibitory
B7-DC	PD-L2/CD273	PD-1 (CD279)	Co-inhibitory
B7-H2	ICOSL	ICOS/CD28	Co-stimulatory
B7-H3	CD276/B7RP-2	TLT-2 (?)	Co-stimulatory/Co-inhibitory
B7-H4	B7S1/B7x	BTLA (?)	Co-inhibitory
B7-H5	VISTA/PD-1H	PSGL-1 (?)	Co-inhibitory
B7-H6	NCR3LG1	NKp30	Co-stimulatory
B7-H7	HHLA2	CD28H/TMIGD2	Co-stimulatory/Co-inhibitory
ILDR2	-	(?)	Co-inhibitory

**Table 2:** Clinical Trials in GC Patients.

Target	Antibody	Combination Treatment	Identifier	Phase	Cancer	Status	Reference
Anti-PD-L1	Avelumab	Irinotecan Paclitaxel	NCT02625623	III	GC	Completed	[187]
Anti-PD-L1	Avelumab	Oxaliplatin 5-FU Leucovorin Capecitabine	NCT02625610	III	GC	Active, not recruiting	[188]
Anti-PD-1 Anti-CTLA-4	Nivolumab Ipilimumab	OTSGC-A24	NCT03784040	I	GC	Recruiting	[189]
Anti-PD-L1	Nivolumab	Radiotherapy	NCT03453164	I/II	GC	Active, recruiting	[190]
Anti-PD-1 Anti-CTLA-4	Nivolumab Ipilimumab	-	NCT03342417	II	GC, Ovarian cancer, Breast Cancer	Terminated (Slow patient accrual)	[191]
Anti-PD-L1	Nivolumab	Tegafur/gimeracil/oteracil Oxaliplatin Capecitabine Placebo	NCT03006705	III	GC	Recruiting	[192]
Anti-PD-1 Anti-CTLA-4	Nivolumab Ipilimumab	chemotherapy	NCT03443856	II	GC, gastroesophageal junction adenocarcinoma	Recruiting	[193]
Anti-PD-L1	Pembrolizumab	Cisplatin 5-FU Capecitabine	NCT02335411	II	GC, gastroesophageal junction adenocarcinoma	Active, not recruiting	[194]
Anti-PD-L1	Pembrolizumab	Cisplatin 5-FU Capecitabine Placebo	NCT02494583	III	GC	Active, not recruiting	[195]
Anti-PD-L1	Pembrolizumab Nivolumab	DSP-7888 Dosing Emulsion	NCT03311334	I/II	Melanoma, GC, Colorectal Cancer, etc.	Recruiting	[196]
Anti-PD-L1	Pembrolizumab Nivolumab	FT500 Atezolizumab Cyclophosphamide Fludarabine IL-2	NCT03841110	I	GC, Colorectal Cancer, Hepatocellular carcinoma, Small Cell Lung Cancer, Renal Cell Carcinoma, etc.	Recruiting	[197]

## Data Availability

Not available.
